# From silos to systems: a retrospective application of the measurement for change (M4C) framework to Grenada's intersectoral early childhood development strategy

**DOI:** 10.3389/fpubh.2026.1955169

**Published:** 2026-10-27

**Authors:** Roberta Evans, Barbara Landon, Toni Murray, Lauren Mohammed, Jesma Noel, Randall Waechter

**Affiliations:** The Caribbean Center for Child Neurodevelopment (CCCN), Windward Islands Research and Education Foundation (WINDREF), Saint George, Grenada

**Keywords:** early childhood development, health equity, intersectoral collaboration, measurement for change, monitoring, evaluation and learning, participatory policy design, small island developing states

## Abstract

**Background:**

Public health systems are often organized around single-sector mandates even when population needs span multiple sectors. Measurement for Change (M4C) was developed within early childhood development (ECD) to position monitoring, evaluation, and learning as a continuous process of adaptation, organized around five aspirations: Dynamic, Inclusive, Informative, Interactive, and People-centered. Whether these aspirations usefully describe national, intersectoral policy development remains largely untested.

**Methods:**

We conducted a retrospective qualitative case study examining the development of Grenada's Intersectoral Early Childhood Development (IECD) Strategy 2026–2030. Over 24 months, the process combined a desk review, 55 key-informant interviews, 34 focus groups, and eight parish-level consultations engaging over 400 participants, together with thematic and SWOT analysis, Theory of Change development, governance design, and costed implementation planning. Qualitative coding combined independent human coding with large-language-model-assisted comparison. M4C was not used prospectively to structure this process; rather, the completed process was examined afterward through the five M4C aspirations to assess convergence, tension, and implications for national-level application.

**Results:**

The process showed strong convergence with all five aspirations, particularly through iterative stakeholder engagement, cross-sector and geographic participation, and translation of evidence into a costed Theory of Change and governance architecture. However, national-level application also exposed limits not evident at program scale. Political favoritism and institutional turf-protection constrained interactivity even where formal governance structures existed. Dynamism depended on institutional capacity to sustain ongoing review and adaptation after external technical support ended. Inclusion required disaggregation fine enough to reveal sub-national inequity. Some politically sensitive priorities, once surfaced, proved harder to act on than to identify.

**Conclusion:**

M4C provides a useful analytic lens for national systems-building, but at this scale its aspirations depend on enabling conditions such as political neutrality, institutional continuity, data harmonization, and decision authority that program-level applications can more readily assume. We offer recommendations for policymakers and researchers applying M4C to national policy design elsewhere, particularly in other small island developing states.

## 1 Introduction

Public health systems are routinely organized around single-sector mandates (e.g., health, education, social protection, environment) even though the problems they exist to solve rarely respect those boundaries. Nowhere is this mismatch more visible than in early childhood development (ECD), where a child's trajectory is shaped simultaneously by health status, nutrition, caregiving quality, protection from harm, and opportunities for early learning (1). Decades of neuroscience and economic evidence have established that the first years of life are a uniquely sensitive period for brain and biological development, and that the returns to investment in this period compound across the life course (2). Yet policy and service delivery for young children continue to be split across ministries that plan, budget, and report independently of one another, producing the duplication, blind spots, and “children falling through the cracks” that fragmented systems predict (3, 4).

Measurement for Change (M4C) was developed as a response to this problem within the ECD field. Emerging from a year-long structured dialogue among ECD practitioners and academics from 21 organizations, M4C reframed monitoring, evaluation, and learning (MEL) as an engine of continuous program improvement rather than an end-of-project verdict on whether an intervention “worked” (5). The approach is organized around five interconnected aspirations for MEL systems: that they be (1) Dynamic, (2) Inclusive, (3) Informative, (4) Interactive, and (5) People-centered (6). A subsequent review of 31 ECD initiatives applying these aspirations across Africa, Asia, Europe, and Latin America and the Caribbean found that M4C's greatest value lay not in any single indicator or tool, but in the discipline of designing measurement so that it continuously feeds back into decisions on the ground (7). This concern echoes a broader public health and public administration literature on the persistent gap between evidence and policy, which similarly finds that data are necessary but not sufficient for policy change, and that translating evidence into decision-relevant, politically legible forms is itself an active and often under-examined part of the policy process (8, 9). This Research Topic extends the M4C lineage from ECD programming specifically into the broader terrain of improving lifelong health and wellbeing (i.e., public health policy), asking whether the same five aspirations can help move policy “from silos to systems” at a national scale.

This paper takes up that question through the development of Grenada's Intersectoral Early Childhood Development (IECD) Strategy 2026–2030. Grenada is a small island developing state (SIDS) in the Eastern Caribbean, comprising the main island, Grenada, and the smaller islands of Carriacou and Petite Martinique. More than half of Grenadian children live in poverty, the highest rate in the Eastern Caribbean (10), and the country's ECD landscape, like that of many small states, combines genuine strengths, including near-universal preschool enrollment, a national Roving Caregivers Programme, and universal health coverage, with the classic symptoms of sectoral fragmentation: no unified data system, inconsistent enforcement of existing ECD standards, and services that are markedly weaker on the sister islands than on the mainland. In 2023, UNICEF's Eastern Caribbean Area Office, in collaboration with the Ministry of Education and the Government of Grenada, asked the Caribbean Center for Child Neurodevelopment (CCCN) to support the design of an integrated, costed IECD strategy. The resulting 24-month process offers a complete, documented record of what it takes to move a national policy system from informal cross-sector cooperation toward an institutionalized, accountable structure.

Our contribution is fourfold. First, we present the Grenada strategy-development process in enough empirical detail to serve as a transferable case study, drawing on the published Strategy document itself (11) and on the underlying qualitative dataset. Second, we map each stage of that process onto the five M4C aspirations, extending the framework's application from program-level MEL to the retrospective analysis of a national, multi-ministerial policy strategy. Third, rather than treating convergence with M4C as a foregone conclusion, we identify specific tensions in the qualitative evidence that are not prominent in M4C's program-level literature, several of which concern political sensitivity, institutional authority, and data integration. Fourth, we distill actionable recommendations, explicitly linked to each M4C aspiration, for policymakers and researchers applying M4C to national policy design elsewhere. In achieving these four goals, M4C was not used prospectively as a formal framework to structure the initial strategy-development process; rather, once the process was substantially complete, we examined the documented process, stakeholder evidence, and resulting policy architecture through the lens of the five M4C aspirations. Thus, this paper does not test whether M4C caused particular features of the Grenada Strategy. Instead, it asks whether an independently developed national policy process converged with the principles M4C articulates, where that convergence was strongest, where national-level application exposed tension or limitation, and what these findings suggest for refining M4C's use in national systems-building. We return to the specifics of this retrospective design, and to the partial exceptions to it, in Section 2.1. Methodologically, this paper also contributes to the M4C literature specifically by describing and validating the use of large-language-model-assisted qualitative coding alongside independent human coding. LLM-assisted qualitative coding is itself an active and growing area of methodological research (12, 13). To our knowledge, it has not previously been applied or validated within M4C-guided measurement practice, where the volume of stakeholder data involved posed a particular challenge for manual coding within a policy-relevant timeframe.

Our guiding question is therefore: to what extent do the five M4C aspirations characterize a national intersectoral ECD strategy-development process, and what does their retrospective application reveal about the utility and limitations of M4C for national systems-building?

## 2 Assessment of policy options and implications: examining M4C in Grenada's national strategy-development process

### 2.1 Analytic approach: M4C as a retrospective lens

For each of the five M4C aspirations, we ask four questions of the qualitative and documentary evidence: (1) What does M4C propose this aspiration should look like in practice? (2) What evidence from Grenada's process converges with that proposal? (3) Where does the evidence instead reveal tension, limitation, or a condition M4C's program-level literature does not anticipate? (4) What does this suggest about applying M4C at national scale? This structure is deliberately designed to avoid treating alignment with M4C as a foregone conclusion. Each subsection below reports convergence and tension together, rather than presenting five confirmations in sequence.

Answering these questions requires a clear definition of what “success” meant for the strategic planning process itself, since without one, any claim that M4C “helped” is unfalsifiable. For the purposes of this case study, we define success in strategic planning not as a downstream change in child development outcomes, which the Strategy's 2026–2030 implementation period has not yet had time to produce, but as the production of a government cabinet-ready, costed, and governed national strategy. The strategy must consist of a defined coordinating body, a resourced implementation plan, and a monitoring and evaluation framework capable of guiding and revising delivery. Grenada's process met this definition of success. The following sections assess where the qualitative evidence shows M4C's five aspirations demonstrably supporting that outcome.

It is important to note that neither the project's Terms of Reference issued by UNICEF's Eastern Caribbean Area Office nor CCCN's Technical and Financial Proposal named M4C or its five aspirations explicitly, and we do not claim that M4C consciously guided the strategy's initial design. However, following the contract award, the CCCN team held internal discussions aimed at ensuring the data-collection instruments reflected M4C-consistent principles before fieldwork began. This intent is reflected in the instruments themselves. The interview and focus group guides were structured around the categories of Mindset, Current State of Affairs, Priorities, and Challenges, with a fifth category, Vision, named explicitly in the interview guide's own coding taxonomy. These match the four categories (priority, challenges, mindset, and vision) later used to structure the 5x4 thematic matrix described in Section 2.3. During strategy development itself, the Theory of Change explicitly named “a measurement for change monitoring and evaluation system that promotes continuous program improvement” as one of its own coordination mechanisms (11). M4C-consistent language thus entered the Strategy's text before this paper was written. M4C's role in this process is therefore best described in three stages. The first is absent by name at the planning stage, though methodologically anticipated. The second is partially and explicitly adopted into the Strategy's own architecture during development. The third is applied in full, as the organizing analytic lens, in the writing of this paper.

Figure 1 summarizes this relationship, showing how the process's stages map onto the five M4C aspirations and how the process returns to earlier stages through iterative review.

**Figure 1:**
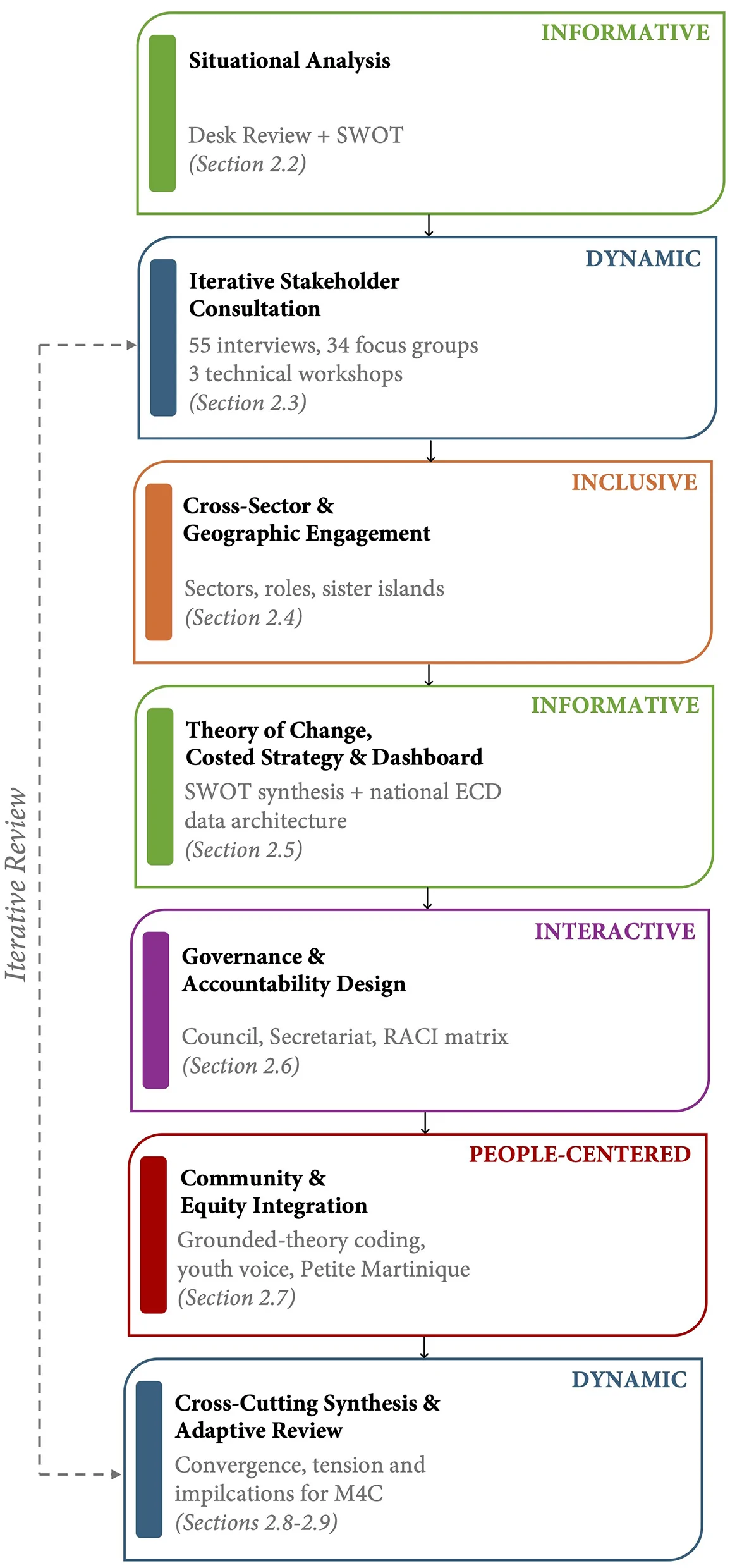
Mapping the stages of the Grenada IECD strategy development process onto their primary M4C aspiration, with iterative review looping back from cross-cutting synthesis into stakeholder consultation.

### 2.2 The starting point: the cost of fragmentation in Grenada's ECD landscape

Before any strategy could be designed, the scale and shape of fragmentation had to be established. A structured SWOT analysis was carried out by synthesizing information collected across one desk review, 55 interviews, and 34 focus groups for the health, education, social development, environment, and intersectoral coordination sectors. This SWOT analysis found several strengths to build on, including near-universal preschool enrollment (approximately 95%), free or low-cost maternal and child healthcare, and informal but consistent cross-sector cooperation among nurses, teachers, and community workers on referrals and outreach. Set against these were weaknesses that will be familiar to public health policymakers well beyond the Caribbean: workforce shortages and undertraining—especially in pediatric mental health and special needs services; fragile, climate-vulnerable infrastructure; a wide and repeatedly cited gap between policy intentions and on-the-ground enforcement. A further weakness most pertinent to this research topic was the complete absence of a unified data system for tracking children's development and service access across ministries. Stakeholders described this fragmentation in consistent language across independent conversations. One official stated plainly, “we are still working in silos... the ministries do not see the link that we are all partners”, while another observed that shared authority was resisted because “they don't want to be monitored, but what gets monitored, gets done.” Threats identified by stakeholders, including climate disasters, workforce migration, donor dependency, and institutional resistance to shared authority, mean that fragmentation in Grenada is an active driver of inequity, particularly for the sister islands of Carriacou and Petite Martinique. This diagnosis is the empirical starting point for everything that follows.

### 2.3 Dynamic: iterative, but not unlimited

M4C's dynamic aspiration specifies that measurement systems remain responsive to an evolving context rather than fixed at a single point in time, following a cycle of collect, review, dialogue, and redesign. The Grenada process converged with this specification during the design phase itself. Following an initial desk review published in January 2025 (14), data collection ran iteratively from June 2024 to May 2025, punctuated by three technical workshops and a preliminary draft-strategy feedback session. It was during the Second technical workshop that emerging findings were shared with stakeholders for validation and adjustment before being included in the Theory of Change. The core interview and focus group question guides remained stable across this period. The synthesis and validation of findings were iterated across the technical workshops.

This dynamism extended to the analytic method itself. To manage the data from 233 interview and focus-group participants and 238 parish consultation participants, the research team used large language models (ChatGPT-4, OpenAI; Microsoft 365 Copilot, Microsoft) alongside manual coding. All data were de-identified before being coded. Two graduate researchers began by independently coding one interview per sector using a grounded-theory approach, producing five preliminary codebooks apiece. Through discussion, these were merged into one consolidated codebook per sector, resolving overlaps and inconsistencies. This calibration step, rather than a *post-hoc* reliability statistic, was the primary mechanism used to establish coding consistency across coders. The coders then split the remaining transcripts and coded them individually, tracking emerging themes in a shared spreadsheet. Sector-level findings were organized into a 5 x 4 matrix crossing the five sectors, Education, Agriculture/Nutrition, Health, Social Development, and Climate/Environment, against the same four overarching categories named in the data-collection instruments themselves (Priority, Challenges, Mindset, and Vision), allowing the team to check for alignment across sectors as well as depth within each one. Because each transcript was coded by only one human coder and the purpose of this stage was to compare human-coded output against LLM-coded output, we did not calculate a formal inter-coder reliability statistic such as Cohen's kappa. To validate the manual process, individual, de-identified transcripts were separately uploaded to the same large language models and analyzed using the prompt “extract priorities (and key words), challenges (and key words), mindsets (and key words), and vision (and key words) from these interviews,” mirroring the manual codebook categories. Outputs were then compared against the independently hand-coded themes for the same transcripts. This comparison found few differences beyond phrasing and how some themes were grouped, and these minor differences were resolved through discussion between the Strategy developers and coders rather than requiring a formal adjudication process.

The qualitative evidence also reveals a clear limit to this dynamism. Iteration was achievable during the design phase, but its sustainability afterward depends on institutional conditions the design phase does not guarantee. One official explained this directly when describing the risk that momentum built during the process could not be maintained: “we may start off very well, but the sustainability factor then becomes an issue... it's impossible to call up a consultant from 2 to 3 years ago who no longer has a contract.” A separate official raised a related concern about staff turnover disrupting institutional memory, noting that personnel central to the process “may be moved at any time... continuity is important.” Dynamism, in other words, was demonstrable as a property of the design process, but its persistence as a property of the resulting system depends on continuity of technical capacity that national institutions, particularly in a small state with a limited specialist workforce, may struggle to guarantee once external technical support concludes.

### 2.4 Inclusive: participation, representation, and political constraint

M4C's inclusive aspiration proposes that a measurement system's data capture the perspectives of those most likely to be missed by conventional monitoring. The Grenada process pursued this on two axes simultaneously: role and geography. On role, engagement extended beyond ministry officials to frontline childcare and protection workers, community health workers, educators, children and youth, key-informant interviews with regional and international partners, and parents—including those of children with disabilities. On geography, eight parish-level consultations ensured that the mainland's own internal diversity was captured, while a dedicated comparative analysis was run between the mainland and the sister islands of Carriacou and Petite Martinique.

That comparison revealed an inclusion failure that national-level aggregate data would have hidden. One participant observed that references to “Carriacou and Petite Martinique” in national planning generally mean Carriacou alone, with Petite Martinique's distinct and more severe vulnerabilities folded invisibly into a shared label. A Petite Martinique participant described the pattern bluntly: “They are always putting us in a box... any government administration, Petite Martinique is like the last of the tri-islands of Grenada. They don't even study us up here.” Consultations on the island also revealed a specific, previously uninvestigated health disparity: residents linked a perceived high local incidence of kidney stones and H. pylori infection to water quality, noting that “no survey has been conducted to understand why” (focus-group participant), and that the island has no resident dentist, requiring routine care to be paid for as an out-of-pocket trip to the mainland. These findings illustrate that disaggregated, place-based measurement can surface not only service-access gaps but potential health disparities that would otherwise remain undocumented.

The evidence also reveals a further constraint on inclusion in that access to information and opportunity was, in some accounts, distributed along political rather than geographic or sectoral lines. One participant described cross-sector activities being withheld from children along partisan lines, explaining that a parent might refuse to let a child participate because “that's their thing, and I don't want to support them.” Another participant described information-sharing itself as “politically motivated,” observing that opportunities circulated among “my friend, your friend, and who we want in our group,” leaving some children without the same access as others. This suggests that inclusive measurement, even when methodologically rigorous, operates within a social context where the equitable distribution of opportunity it aims to document is not guaranteed by data collection alone.

### 2.5 Informative: evidence translated into decisions, requiring integration

M4C's informative aspiration specifies that measurement be designed to answer decisions that matter rather than simply describing a situation. In Grenada, the thematic and SWOT findings were converted into a formal Theory of Change with an explicit problem statement, causal pathway, medium-term outcomes, short-term outputs, and named assumptions and risks for each output. The Theory of Change was co-developed during a participatory technical workshop facilitated by CCCN, with technical guidance from Monitoring and Evaluation and Early Childhood Development Specialists from UNICEF's Eastern Caribbean Office and active input from government and stakeholder representatives. Notably, Output 1.1 of that Theory of Change names, as one of its four defining components, “a measurement for change monitoring and evaluation system that promotes continuous programme improvement” (11). The Strategy adopted M4C's own language and logic as one of its four core coordination mechanisms, alongside a costed implementation plan, a shared intersectoral data system, and a cross-sectoral referral system.

This aspiration was operationalized concretely under Strategic Objective 3: the digitization of cumulative child records as an extension of the Ministry of Education's existing Education Management Information System. This digitization integrates relevant data from the Ministries of Education, Health, Social Development, and Mental Health, the Child Protection Authority, and the Grenada Food and Nutrition Council. It includes the training of 700 personnel on data entry and the construction of a national ECD data dashboard. The Strategy's overall projected annual cost of XCD $2,668,847 is expressed as a transparent figure of XCD $222.40 per child aged 0–8 annually, a framing chosen explicitly to support legislative and public accountability.

The qualitative evidence, however, indicates that this cross-ministry data integration is considerably harder to achieve than to specify. One official described a concrete instance of the problem the dashboard is designed to solve: “it is almost impossible to get numbers at that level from the ministries... Even when you get it, it is not always accurate.” A separate official connected this directly to the mechanics of measurement itself, noting that without reliable inter-agency follow-up systems, “a lot of the things we are hoping to measure through the framework become difficult to report on at all.” Informative measurement requires not only a technical architecture for integrating data, which Grenada's Strategy specifies, but harmonized definitions, trusted data-sharing norms, and sustained inter-agency cooperation. These are conditions the evidence suggests do not yet fully exist.

### 2.6 Interactive: from consultation to institutionalized authority

M4C's interactive aspiration concerns whether data actually provoke dialogue and decisions. In Grenada, the governance structure ultimately recommended a National Intersectoral ECD Council with civil society representation, a national ECD Coordinator and Assistant Coordinator, and an implementing Secretariat reporting through the Minister for Education to Cabinet. This governance structure emerged directly from a stakeholder-identified priority (“Governance & Structure”) that surfaced repeatedly across the desk review, consultations, and technical workshops. The Strategy operationalizes this beyond the design phase through a responsible-accountable-consulted-informed (RACI) matrix (15), converting interactivity from a one-time feature of consultation into a standing operating rule intended to persist through the Strategy's implementation period.

The qualitative evidence indicates that this institutionalization responds to a real and specific problem identified by interviewees. When asked directly why intersectoral collaboration has historically been difficult, participants offered concrete, converging answers: “inefficient communication,” unclear role definitions, workers becoming “numb” to reported issues, and confidentiality concerns acting as a deterrent to reporting across agencies. Institutional actors also described active resistance to shared accountability. One official, explaining why a cross-ministerial oversight mechanism was historically avoided, stated that ministries “don't want other ministries to be in their business... they don't want to be monitored, but what gets monitored, gets done,” attributing this to “silos and the turf war.” Frontline workers echoed this in their own terms, describing sectors as “insular” and observing that professionals “guard their specializations because that is their competitive advantage.” Stakeholders also flagged a risk the RACI matrix alone cannot resolve: one participant cautioned that the new coordinating body “should be away from politics... but it's hard to not be biased.” Interactive governance, at national scale, therefore appears to require not only institutionalized rules for who is consulted and who decides, but active management of the political and professional incentives that make institutions reluctant to be monitored in the first place.

### 2.7 People-centered: voice, equity, and the limits of participation

M4C's people-centered aspiration asks that measurement keep children, families and communities, rather than institutions, at its center. The Grenada process pursued this by identifying, through the SWOT analysis, community and cultural assets, extended family support, communal caregiving traditions, and grassroots leadership as strengths to recognize and build upon rather than informal stopgaps to be replaced by new parallel systems. The grounded-theory coding approach used to validate the thematic analysis began, by design, with participants' own language and insights before any external framework was applied, and the published Strategy retains direct stakeholder quotations throughout its findings sections.

Youth participation in particular exceeded what is typically documented in national policy processes of this kind. In dedicated youth focus groups, young people raised sophisticated, systems-level observations in their own words, independently naming “silos” and “resistance to change” as barriers to effective service delivery, and offering a detailed critique of the child protection and foster care system's institutional trustworthiness. This is notable because a common critique of people-centered measurement is that children are consulted as informants but rarely treated as measurement actors in their own right. The specificity and system-level character of the youth data gathered here suggests this concern, while generally valid, does not fully describe what occurred in Grenada.

At the same time, the evidence reveals a genuine tension between people-centered specificity and the demands of national standardization. During a Theory of Change validation session, a senior stakeholder explicitly cautioned the team against assuming that interventions could be uniform across the mainland, Carriacou, and Petite Martinique: “we need to figure out if the interventions must be the same... If you do not know my needs, you cannot help me in the best way”. The team also received explicit guidance on the practical difficulty of achieving genuine, rather than token, community engagement with historically marginalized communities: “they become closed off and standoffish... don't show your hand so early.” People-centered measurement, in other words, was achievable in the sense of gathering rich, specific, community-grounded evidence, but translating that specificity into a single national strategy required active, ongoing judgment about how much local variation a standardized policy architecture can actually preserve.

### 2.8 A cross-cutting pattern: political sensitivity as a constraint across sectors

One finding recurred across sectors clearly enough to warrant separate attention, since it does not map neatly onto any single M4C aspiration but appears to constrain several simultaneously. Certain substantive topics were identified as measurement priorities but proved difficult to translate into action at the same pace as others, specifically where they intersected with religious, cultural, or political sensitivity. One interviewee described corporal punishment and gender-related policy content as material that, if pushed too directly, risked becoming a “poison pill” capable of “chasing partners away from the table” given anticipated resistance from religious and community stakeholders. A separate interview, in the environment and climate sector, described an almost identical dynamic: “it's similar to how we approach gender issues at arm's length... climate change is treated in much the same way... we don't talk about it at all.” Taken together, these two independent accounts, drawn from different sectors and different interviews, suggest that inclusive and informative measurement can successfully surface politically sensitive priorities without the surrounding political process necessarily being equipped to act on all of them at the same pace. This is a limitation on what measurement itself can achieve, rather than a methodological shortcoming in how the measurement was conducted.

### 2.9 From silos to systems: synthesis

Beyond service-delivery coordination, the Strategy's Theory of Change was also mapped explicitly to the WHO/UNICEF/World Bank Nurturing Care Framework (2), the Sustainable Development Goals (16), and Grenada's own National Sustainable Development Plan 2020–2035 (17). This extended the systems logic documented in Sections 2.3 through 2.8 from service delivery coordination to alignment with global and domestic policy architecture.

Taken together, the aspiration-by-aspiration evidence in Sections 2.3 through 2.8 suggests that Grenada's process converged substantially with all five M4C aspirations while also revealing that each aspiration depends, at national scale, on enabling conditions that program-level applications of M4C can more readily take for granted. Dynamic measurement was achievable during design but depends on institutional continuity to persist. Inclusive measurement successfully surfaced sub-national inequity but operates within a political environment that does not always distribute opportunity equitably regardless of what the data show. Informative measurement produced a costed, decision-ready Theory of Change but depends on cross-ministry data integration that does not yet fully exist. Interactive governance was institutionalized through a proposed Council, Secretariat, and RACI matrix, but these formal structures address, without fully resolving, an underlying incentive for institutions to resist the visibility that accountability requires. People-centered measurement produced rich, specific, community-grounded evidence, including substantive youth voice, but translating that specificity into a single national strategy required continuous judgment about how much local variation a standardized policy can preserve. Table 1 summarizes this pattern of convergence and tension across all five aspirations, together with the implication each carries for applying M4C at national scale.

**Table 1:** Convergence, tension, and implications of the five M4C aspirations in Grenada's national IECD strategy-development process.

M4C aspiration	Convergence with Grenada Evidence	Tension or limitation	Implication for M4C at national scale
Dynamic	Iterative desk review, interviews, and technical workshops with repeated validation cycles during design.	Sustaining iteration depends on institutional capacity and continuity of technical expertise after external support ends.	Dynamism should be assessed as a property to be resourced and sustained, not only as a property of the design phase.
Inclusive	Engagement across sectors, roles, and geography, including dedicated sister-island consultations that surfaced Petite Martinique's distinct, previously hidden needs.	Access to information and opportunity was, in participant accounts, sometimes distributed along political rather than equitable lines.	Inclusive measurement should be evaluated by whether opportunity is equitably distributed in practice, not only by whether data collection was broad.
Informative	SWOT and thematic analysis converted into a costed, decision-ready Theory of Change with a defined data architecture (Strategic Objective 3).	Cross-ministry data integration required by the architecture is not yet consistently achievable; officials described real, present difficulty obtaining reliable inter-agency data.	Informativeness at national scale should be treated as contingent on data-harmonization capacity, not assumed once a technical architecture is specified.
Interactive	A stakeholder-identified governance priority produced a Council, Secretariat, and RACI matrix defining ongoing accountability.	Institutional actors described active resistance to shared accountability (“turf war”) and a risk of political interference in the new coordinating body.	Institutionalizing interaction addresses, without fully resolving, underlying incentives against the visibility accountability requires.
People-centered	Grounded-theory coding beginning from participants' own language; substantive, systems-level youth input; family and community consultations.	Sophisticated stakeholder guidance cautioned against assuming uniform interventions across the mainland and sister islands.	People-centeredness at national scale requires ongoing judgment about how much local variation a standardized strategy can preserve.

## 3 Actionable recommendations

The Grenada experience suggests that applying M4C at the national systems level requires translating its five aspirations into explicit design requirements and explicit safeguards against the tensions identified in Section 2, rather than treating them as general characteristics of a good measurement system. We offer the following recommendations for policymakers, technical partners, and researchers applying M4C to national, cross-sectoral public health strategy design elsewhere, particularly in other small and resource-constrained states.

### 3.1 Dynamic

Resource iteration as an ongoing institutional function, not a design-phase activity. Build predefined, funded opportunities for evidence review and revision into the strategy's implementation period itself, with named institutional ownership that does not depend on externally contracted consultants, so that dynamism can survive the end of technical assistance.

### 3.2 Inclusive

Measure whether opportunity is distributed equitably, not only whether consultation was broad. Disaggregate to the smallest meaningful geographic and demographic unit, since national or regional aggregates can mask acute exclusion, and pair this with explicit monitoring of whether services and opportunities reach communities equitably regardless of political affiliation or geographic remoteness.

### 3.3 Informative

Invest in cross-ministry data harmonization before, not after, specifying a shared architecture. Establish common data definitions, validated data-sharing agreements, and named data stewards across ministries as a precondition for a national dashboard or shared data system, since the technical architecture alone does not resolve the inter-agency trust and consistency problems that generate unreliable cross-ministry data.

### 3.4 Interactive

Pair institutional accountability mechanisms with explicit safeguards against political interference and institutional resistance. Name accountability explicitly, for example through a RACI matrix, but treat this as necessary and not sufficient; anticipate and plan for resistance from institutions that experience monitoring as a loss of autonomy, and build explicit protections against the politicization of coordinating bodies into their governing terms of reference.

### 3.5 People-centered

Create structured space for local and youth voice within a standardized national architecture. Treat community and cultural assets, and youth perspectives specifically, as strategic infrastructure rather than informal background, while building explicit mechanisms, such as differentiated implementation guidance by geography, to preserve locally meaningful variation within a nationally standardized strategy.

### 3.6 Across all aspirations

Name politically sensitive priorities explicitly rather than allowing them to be quietly deprioritized. Where measurement surfaces politically or culturally sensitive findings, such as those concerning gender, corporal punishment, or climate change in this case, build an explicit, staged plan for addressing them rather than allowing their absence from early implementation phases to go unacknowledged.

### 3.7 AI-assisted analysis

Pair human coding with large language model (LLM) analysis, and validate the pairing transparently. Where large qualitative datasets exceed available technical capacity use LLMs to accelerate thematic and SWOT analysis, but validate a meaningful proportion of the output against independent, grounded-theory human coding, and report explicitly which reliability checks were and were not conducted before relying on the output for policy decisions.

These recommendations include potential risks. Recommendation 6, naming politically sensitive priorities explicitly, carries a genuine risk of becoming self-defeating. The same visibility that allows a sensitive priority to be planned for could also trigger the very resistance described in Section 2.8, if raised without adequate groundwork or political timing. Recommendation 4, calling for institutional safeguards against politicization, assumes a degree of political will to constrain political behavior that may not be available in every context, and could itself become contested. Recommendation 3, calling for cross-ministry data-harmonization, requires real time and resources that compete directly with more visible, immediate service-delivery priorities, and a government under fiscal or political pressure may reasonably deprioritize it. Conversely, the tensions cataloged in Table 1 offer a reasonable basis for anticipating what is likely to persist, or worsen, if these recommendations are not adopted. These include interactive governance structures that lack explicit protection against political capture, informative systems that remain unable to reconcile data across ministries, and inclusive processes that continue to distribute opportunity unevenly regardless of how broadly consultation is conducted. We offer these recommendations, in other words, as a reasoned position on balance, rather than as a costless or self-executing prescription.

## 4 Discussion

This paper's central contribution is to extend Measurement for Change, developed and previously applied primarily at the level of individual ECD programs and interventions, to the retrospective analysis of a national, multi-ministerial policy strategy. In doing so, it responds directly to this Research Topic's shift in focus from ECD-specific MEL collections toward the broader question of what M4C can offer public health policy design generally. Unlike an account that treats M4C's fit as self-evident, the evidence presented in Section 2 suggests a more conditional claim. The five aspirations translate meaningfully to national scale, but their meaning shifts once the unit of change becomes a multi-ministerial system rather than a single program. At program level, M4C asks whether measurement is dynamic, inclusive, informative, interactive, and people-centered in relation to implementation and learning. At national level, the same aspirations must additionally contend with institutional authority, competing sectoral mandates, political incentives, and the translation of evidence into collective, cross-ministerial decisions.

This case also speaks directly to established theories of collaborative governance and implementation science. The National Intersectoral ECD Council, Secretariat, and RACI matrix described in Section 2.6 closely mirror what collaborative governance theory identifies as core conditions for effective cross-sector institutions, including shared ownership, face-to-face dialogue, and trust-building among diverse stakeholders (18, 19). Likewise, the iterative, feedback-driven design process mirrors core principles from implementation science, in which the fit between an intervention and its context is treated as something to be continuously assessed and adjusted rather than fixed in advance (20, 21). The tensions identified in Section 2, however, particularly institutional resistance to shared accountability and the political sensitivity of certain priorities, suggest that collaborative governance and implementation science do not merely reinforce M4C's aspirations but also help explain why those aspirations are harder to realize at national scale than at program scale. National systems concentrate more institutional actors with more to lose from increased visibility.

This case also resonates with the health systems strengthening literature, which similarly frames systems as interconnected building blocks whose performance depends on how well they are coordinated rather than resourced in isolation (22–24), and with the emerging concept of learning health systems, in which routine data collection is designed explicitly to drive continuous organizational learning rather than retrospective accountability alone (25). It also builds on a broader evidence base establishing early childhood development as a foundational, cross-sectoral investment with lifelong returns (26, 27), reinforcing why an intersectoral, measurement-driven approach is particularly well suited to this domain, even as Section 2 shows that suitability in principle does not eliminate the practical difficulty of achieving it.

### 4.1 Limitations

Several limitations must be considered when attempting to generalize our findings. This is a single-country, single-strategy case, developed in a small island developing state with a comparatively small population, a high density of technical assistance and donor relationships, and a tri-island structure that made sub-national comparison unusually tractable. Larger or more federated public health systems may find the same aspirations harder, though not impossible, to operationalize at scale. The validation of AI-assisted coding, while a valuable methodological contribution, covered less than half of the qualitative dataset, relied on tools and model versions that are evolving rapidly, and used a prompt that was refined iteratively during analysis rather than fixed and pre-registered in advance. Given that each transcript was coded by only one human coder rather than by both, we did not calculate a formal inter-coder reliability statistic. This was a deliberate design choice given that this stage was intended to compare human-coded output against LLM-coded output rather than to cross-validate two independent human coders. Future applications of this method would benefit from a pre-registered prompt protocol and a quantitative agreement statistic. Because the Strategy was published in December 2025, this paper describes a design process and its intended architecture. It cannot yet report on implementation fidelity, dashboard uptake, or downstream child development outcomes, and the projected cost and return-on-investment figures are projections rather than realized results.

A further limitation concerns potential bias in the research process itself. The research team, based at CCCN, both facilitated the strategic planning process and led the retrospective analysis presented here, creating a risk that findings were interpreted in ways that favored a positive account of a process the team had itself designed and delivered. We sought to offset this through the use of independent graduate coders, grounded-theory coding that began from participants' own language, and by deliberately structuring Section 2 to report tension alongside convergence for every aspiration. Stakeholder consultations were also subject to selection bias. Participants were identified through government and CCCN networks and were, by definition, willing and available to take part, which may under-represent the perspectives of families and frontline workers who were hardest to reach or most disengaged from formal ECD services. Finally, the thematic and SWOT analyses were conducted by a team already familiar with M4C by the time of analysis, which raises the possibility of confirmation bias in how qualitative findings were categorized and interpreted. The parallel large-language-model coding described in Section 2.3 was one attempt to mitigate this, but it does not eliminate it, since both the human coders and the prompts given to the models were shaped by the same underlying analytic framework.

### 4.2 Transferability

Although developed in Grenada, the approach described here offers a transferable model for other countries seeking to examine or operationalize Measurement for Change in national policy. Rather than prescribing a fixed sequence of activities, the process demonstrates how iterative stakeholder engagement, theory-driven measurement, structured governance design, implementation planning, and adaptive monitoring can be integrated into a coherent policy development pathway, while also demonstrating that this integration is neither automatic nor free of political constraint. These principles are likely to be applicable across diverse settings, particularly in low- and middle-income countries and other small island developing states seeking to strengthen intersectoral governance. It is important, however, to distinguish which elements of this pathway depend on Grenada's scale and which do not. The speed of Grenada's coordination benefited directly from SIDS-specific conditions. These include a small number of ministries, a handful of key decision-makers who could be convened directly and repeatedly, and a national government with the standing authority to establish a single, Cabinet-ready coordinating body without navigating multiple sub-national layers of government. In a larger or more federated system such as a country with state- or provincial-level authority over health and education, the same M4C aspirations would likely still apply, but achieving them would require additional coordination layers, longer consensus-building timelines, and governance structures capable of reconciling national and sub-national authority. What is more generalizable, independent of scale, is the underlying logic that measurement should be embedded in governance design from the outset rather than added afterward. Additionally, iterative and disaggregated data collection can identify sub-group exclusion that aggregate reporting would miss. Further political and institutional resistance to shared accountability is likely to recur wherever M4C is applied to systems with more than one institutional owner. As a single-country case study, this paper is not positioned to fully specify what additional structures, coordination layers, or timelines would be necessary to achieve the same outcomes in a country with more complex, multi-level, or federated governance and health systems. This is a potential direction for future comparative work across SIDS and non-SIDS contexts. Finally, it is important to note that this architecture is necessary but not sufficient on its own. Stakeholders themselves named risks such as donor dependency, political turnover, workforce migration, and recurrent climate shocks, all of which could undermine well-designed and sustained political commitment to an intersectoral ECD strategy.

## 5 Conclusion

This paper set out to ask whether the five M4C aspirations usefully characterize a national, intersectoral ECD strategy development process, and what their retrospective application reveals about M4C's utility and limitations at this scale. We find substantial convergence. Grenada's process was iteratively designed, broadly inclusive, translated evidence into a costed and governed strategy, institutionalized feedback through formal governance structures, and grounded its analysis in participants' own language. We also find that convergence was neither automatic nor complete. Political favoritism and institutional resistance constrained interactivity even where formal structures existed, cross-ministry data integration remained aspirational rather than achieved, dynamism proved easier to establish than to sustain, and some politically sensitive priorities proved harder to act on than to surface. M4C's aspirations, in other words, provided a genuinely useful analytic lens for understanding this process, but the process also reveals dimensions, such as political neutrality, institutional continuity, data harmonization, and decision authority, that the framework's program-level literature does not fully anticipate and that we suggest deserve greater emphasis when M4C is applied to national systems-building. Future research should follow this case forward, prospectively evaluating whether Grenada's IECD dashboard and Council structure are used as designed, and whether they in turn shift resource allocation and child outcomes over the Strategy's 2026–2030 period. Comparative work applying the same aspiration-by-aspiration approach used here to other small island developing states, and to public health domains beyond early childhood development, would help establish how far M4C's applicability, and the tensions identified here, extend beyond this case.

## Data Availability

De-identified participant data that support the findings of this study are available from the corresponding author upon reasonable request and subject to approval by the St. George's University Institutional Review Board to protect participant confidentiality. The study's main findings and the full strategy document are publicly available in the Grenada Intersectoral Early Childhood Development Strategy 2026–2030, which is accessible on the Caribbean Center for Child Neurodevelopment website (www.cccnd.org). Please contact the corresponding author for further access details.
